# A Push on Job Anxiety for Employees on Managing Recent Difficult to Understand Computing Equipment in the Modern Issues in Indian Banking Quarter

**DOI:** 10.1155/2015/839216

**Published:** 2015-10-07

**Authors:** Ragunathan Gopalakrishnan, Chellapa Swarnalatha

**Affiliations:** Department of Management Studies, Anna University Regional Centre, Madurai, Tamil Nadu 625 002, India

## Abstract

Stress management can be defined as intervention planned to decrease the force of stressors in the administrative center. These can have a human being focus, aimed at raising an individual's ability to cope with stressors and the implementation of the CRM is essential to establish a better performance of the banking sector. Since managing stress and customer relationship management are becoming crucial in the field of management the work has forecasted them in a wide range of dimensions. This paper organizes few preliminary concepts of stress and critically analyzes the CRM strategy implemented by banking sector. Hence the employees of the Banking Industry have been asked to give their opinion about the CRM strategy adopted by banks. In order to provide the background of the employees, the profile of the employees has been discussed initially. The profile of the employees along with their opinion on the CRM practices adopted at Banking Industries has been discussed. In our work progresses we have been taken of two main parameters for consideration and it detriment in which area stress are mainly responds, and also the paper envelopes certain valuable stress management tactics and techniques that are particularly compassionate for people who have been working in the banking sector. Also an attempt to diagnose the impact of underside stress of day to day life in mounting a bigger level stress upon the employees has been made. Further development has been made with a detailed parametric analysis of employee stress conducted with the wide range of key parameters and several rounds of experiments have been conducted with techniques as Kolmogorov-Smirnov test, Garrett ranking, and ANOVA; the work ensures to pave way for an accurate measure in customer handling. The questionnaire is planned to be distributed to 175 employees in the Madurai district banks.

## 1. Introduction

The implementation of the CRM is essential to establish a better performance of the Banking Industry and to avoid stress of employees. The present study critically analyzes the CRM strategy implemented by Banking Industry and find out the stress level of employees. Hence the employees of the Banking Industry have been asked to give their opinion about the CRM strategy adopted by banks. In order to provide the background of the employees, the profile of the employees has been discussed initially. In this research article, the profile of the employees and their opinion on the CRM practices adopted at Banking Industry has been discussed. The paper deals with the systematic presentation of analyzed data followed by the interpretation of data. Statistical analysis of data enables researchers to organize, evaluate, interpret, summarize, and communicate numeric information. Descriptive statistics is used to describe data and inferential statistics to draw inferences about a population based on collected sample data.

Banking sector not only has been playing a leading role within the financial system in India but also has a significant socioeconomic function, making intruding into the interiors of the economy and is being considered as one of the fast developing areas in the Indian financial sector too.

Customer service is an integral part of Banking Industry but also while handling the number of customers the stress level will automatically increase. It is the duty of research to do in-depth study to identify the key success factors in Banking Industry, in terms of stress level of the employees so as to survive in intense competition and increase the market share.

CRM is a customer focused business strategy that aims to increase customer satisfaction and customer loyalty by offering a more responsive and customized service to each customer. CRM is about managing customers for better understanding and to serve them. Keeping the importance of CRM and its service excellence in view, this study is an attempt to analyze management of the customer relationship in banking sector particularly, Banking Industry of India (banks) in Madurai district of Tamil Nadu.

## 2. Objective Is to Analyze Influence of the CRM Factor

- To examine the relationship between employee's job stress and their involvement in organizational works which in turns in CRM.
- To examine and understand how the employees know about CRM strategy in bank.
- To analyze the most important category of customers approaching the employees.
- To analyze the satisfaction level of the customer services provided by Banking Industry and find out the occupational status of the employees.
- To find out and analyze the opinion about the CRM system in an integral part of the work in banks.
- To analyze and find out stress level while solving the problem of the account. holders and clearing the doubts of the account holders regarding loan details and other operations.

## 3. Need for the Present Study

Banking sector in India is presently witnessing a phenomenal growth. We presently have over 27 public sector nationalized banks, 31 private banks and 38 foreign banks. All these banks cater to over 114014* *ATMs across the country. But the average population per bank branch (APBB) as on March, 31, 2013, is only 12,100. This means that minimum numbers of banks are catering to a huge volume of people and the growing economy puts the stress right on the shoulders of the bank employees which makes the current study an inevitable one. Although several researches have been made in this area, this work tries to bring in a multidimensional study on the stress in the banking setup.

## 4. Literature Review

Workplace stress places significant psychological, physiological, and financial costs on both the individual employee and his or her organization. Workplace stress has been associated with the etiology of physical disorders such as heart disease, hypoadrenia, immunosuppressant, and chronic pain. In addition, the psychological impact of workplace stress includes depression, persistent anxiety, pessimism, and resentment. The impact of these symptoms on organizations is significant as these symptoms lead to hostility in the workplace, low morale, interpersonal conflict, increased benefit expenses, decreased productivity, and increased absenteeism (Colligan and Higgins [1]).

Job stress can arise from different environment of work like organizational or situational stress; it is from the characteristics of the workers themselves, that is, dispositional stress Riggio et al. [2]. Stress is a natural lesson in the life and every employee, even executives and managers, should be affected by this issue. According to survey about 100 million workdays are being affected due to stress problem among employees and nearly 50%–75% due to disease cause stress Bashir and Ramay [3].

Absence and loss of employment are major causes of job stress in the organization. The ratio increases day after day because of organisation environment. They were the main hurdles of achieving goals and performance [4].

Employers need to be aware of how the population (organization) is changing with respect to age. For example, the new trends in the banking industry show an inclination towards more hiring of young and fresh business graduates. So, in near future, in most jobs, even top-level executives would be young people. This also poses another issue that young individuals are more aggressive and sensitive so they are more likely to fall prey to job stress, Du et al., [5].

Ho et al. [6] investigate the relationship between compensation benefit of employees on the basis of performance. Compensation relates to performance of employee in the organization and accordingly shows high performance as well as low performance of the employee.

Income has a major impact on the living standard of an individual. In reality, if it is said that it is the decider of the life-style of any individual, it would not be wrong. Income has also relationship with family life cycle which actually moulds the spending pattern of a family. Different researches have shown that the person with high income is having a different style of spending than low income groups' persons. If a person has a family to support and the number of households is large, then his only criteria of selection of a job would be the money which he would receive. So, any such individual who is being paid less while his expenditure is more would eventually experience job stress, Kanagaretnam et al. [7].

Adding to the pressures that workers face are new bosses, computer surveillance, fewer health, and retirement benefits, and the feeling they have to work longer and harder just to maintain their current economic status. Workers at every level are experiencing increased tension and uncertainty. They are undergoing Job Stress to be precise. This study examines the relationship between Job Stress and Employee Retention and consequences of high stress on Indian Industries.

Shah et al. [8] culture create society and exchange views. It changes from generation to generation. Human nature changes fill the individual gap.culture changes individual behavior also. The symbols may be intangible (attitudes, beliefs, values, and language) or tangible (tools, housing, products, and works of art). Cultures do change over time. Every organization has its own distinct culture. If an employee fails to comply with the organizational norms and culture, he would be proving himself to be odd against all so he would be more prone to have stress at his workplace.

Bano and Kumar Jha [9] analyzed the impact of organizational stress among the public and private sector employees and found that both of them face reasonable levels of stress in work environment. There is no significant difference in terms of total stress levels and work experience but educational qualifications contribute certain differences. In the study by Vishal et al. [10], employees of both public sector and private sector banks face the same level of stress. Among the various stressors in the public sector banks, lack of efficient manpower and performance pressure play major roles. The other contributing factors include family demands and unexpected contingencies followed by job rigidity. The employees of the private banks also face the same stressors along with certain new aspects like adaptability to change and performance pressure which also have some effect on stress.

Joshi and Goyal [11] studied the exposure of stress during the mergers of Bank of Rajasthan with the ICICI Bank. Due to this kind of mergers, both banks create a positive effect on their market base and ensure that the customers of the bank are satisfied. During the merger, the employees of the Bank of Rajasthan were totally against the merger process. Their research found that after the completion of merger the employees satisfaction levels were very low and the stress was very high. They discussed the cultural fit and the human resource policy framework during the merger and felt that if mergers and acquisitions were not managed properly the stress at workplace will increase rapidly. According to a recent study conducted by Dr. Samartha et al. [12], the entry of private and multinational banks, the public sector banks were forced to introduce new concepts and revise their existing setup and product line-ups. This forced the employees of the public sector banks to work beyond their office hours. Hence, the stress levels are at par with the private bank employees.

Upon analyzing the employee turnover in banking sector, Shukla and Dr. Sinha [13] found that the important factors for employee turnover are effective job satisfaction and conducive work environment. The significance level for both these variables are very high and they imply that, irrespective of better salary compensation, the employees have a huge desire to push themselves into new avenues so as to seek a better work environment and job satisfaction. They suggested that if the career-oriented employees were given more freedom and opportunity in the work environment they may not switch companies in a short time. Callous management attitude and excessive workload will stress the career oriented employees and push them to seek better alternatives. Shahid et al. [14] discussed the six components of job stress: lack of administrative support, excessive work demand, problematic customer relations, coworker's relationship, family and work life balance, and riskiness of job. They studied the relationship between stress and performance. The results depicted that stress naturally affects employee performance.

Job stress leading to a counterproductive work behavior has been discussed by Aftab and Javeed [15]. Various factors like poor communication skills, forcing the employees to work beyond their capacity, an unfair performance evaluation system, inappropriate salaries, and working conditions have been analysed. Their findings have proved that job stress leads to counterproductive work behavior thereby affecting the work environment. Sharma et al. [16] discovered that the commercial banks are required to wake up to the fact that stress has multifaceted relationship with performance-related benefits. Performance benefits can lower the perception of underutilization at workplace. On the other hand, it may increase the workload of employees. Nevertheless, an organization aims at reducing the role stress plays at workplace. It involves an uphill task in optimizing utilization of the capabilities of the workforce and at the same time not increasing the workload of employees beyond a functional level.

Shah and Hasnu [17] identified the key factors that have effect on employees' job performance. Job instability is one of the reasons for job stress among employees. This in turn affects the job performance. This study is conducted to understand the causal relationship between job instability and job performance. It aims to identify the key moderators of job performance in the banking sector of Pakistan. Six hypotheses are tested using AMOS 17.00 by applying the technique of structural equation modeling. The findings revealed that all the hypotheses are accepted with significant level. It is proven that there exists a negative relationship between job performance and job stress as well as job instability. This would mean that increase in job instability and job stress would decrease job performance. Job involvement has shown a significant and positive impact on job performance. The direct and indirect relationship showed that all the three variables are the mediators of job performance. Removing job instability and job stress is impossible but organizations can make efforts to reduce them to the minimum level.

Abdullah et al. [18] found that the employees who are extroverts, that is, the people who are more talkative and social, outperform the introverts, that is, who are confined to their own self. Similarly, the employees who were cooperative with their coworkers and superiors perform better than the noncooperative ones. Their study has suggested that proper personality analysis has to be performed before recruiting banking professionals. Adjei and Kwasi Amofa [19] assessed the job related stress at the Barclays Bank and found out that the majority of the employees felt huge stress at work. Their results showed that the long working hours contributed to the stress. Various stress relaxation techniques were suggested to overcome the negative aspects of stress.

## 5. Tool of Data Collection and Research Question

Statistical Package for Social Sciences version 20.0 (SPSS) was used in the analysis and interpretation of data. Two public sector banks and two private sector banks were considered for the purpose of this research. A well-structured questionnaire containing 39 questions leading to 131 variables was used to collect data. The questionnaire was distributed to the banking employees directly and testing was survey primarily conducted from 131 numbers of respondents. The particular information was composed of the banking employees at all three levels in banking sector. Interviews were conducted with the employees for assembly of the various sequences on their observation about their bank (association) and the troubles which they face both directly and indirectly while completing every day jobs. The respondents were interviewed on the issues touching the stress levels of the workers, bang of relations pressures on their employment, expectations from their responsibility, up to what level they are happy, and likely suggestions for overcoming the adversities of stress by evaluating the individual initiatives and managerial initiatives.

## 6. Analysis of Data

The data will be analyzed to determine any differences between the stress levels of employees and their impact on reducing stress. So the researcher attempts to analyze the demographic profiles such as gender, age, sex, marital status, qualification, family size, occupational status, monthly income, association with Banking Industry, number of new account openings, and sectioning of loans are taken for the mode of the selected customers. For the purpose of analysis, 110 employees were taken into consideration.

## 7. Findings and Discussions

- This paper also includes an analysis of data collected by representing it in tabular form along with interpretations.
- The information collected was analysed for arriving at proper conclusion on the topic.

In order to understand how the employees know about CRM strategy of bank employees Garrett ranking technique was employed and the results are given in Table 1.

### 7.1. Garrett Ranking Technique

Table 1 shows details about how the employees came to know about CRM strategy of Banking Industry in Madurai district. It is inferred that orientation given by officials ranked first, which represents a mean score of 58.86, through training ranked second, which represents a mean score of 57.73, through journals and magazines ranked third, which represent a mean score of 48.5, through colleagues ranked fourth, which represents a mean score of 45.95, and personal experience ranked fifth which represents a mean score of 38.95.

The most important category of customers approaching the employees includes farmers, businessmen, professionals, government employees, and private employees. The distribution of employees on the basis of category of customers approaching them is given in Table 2.

The analysis infers that the most important category of customers approaching the employees is professionals and businessmen which constitute 36.36 per cent and 31.82 per cent of the total, respectively.

The most important stages at which the customers are approaching the employees are classified to at the time of opening account and making deposit. The distribution of employees on the basis of the stages at which the customers are approaching is given in Table 3.

It is revealed from Table 3 that the most important stage at which the customers are approaching the employees is at the time of opening new account and long term deposit is 33.64 per cent, at the time of loan repayment 28.18 per cent, and at the time of getting loan 27.27 per cent to the total, respectively.

The satisfaction with the customer services provided by the Banking Industry may also determine the perception level of employees towards CRM in banking sector. The distribution of employees on the basis of satisfaction with the customer services provided by banking is given in Table 4.

Table 4 shows that 76.36 per cent of the employees are satisfied with the customer service provided by the banks while the remaining 23.64 per cent of the employees are not at all satisfied with the service provided by the Banking Industry.

The most important reasons how the employees are satisfied with the services provided by banks are classified into quick response, innovative service delivery, building relationship, good rapport, and financial security. The distribution of employees on the basis of how they are satisfied with the services provided by banks using Garret ranking technique is given in Table 5.

On the basis of the ranks assigned by the employees the impacting variables are analyzed through Garrett ranking techniques. It is evident from Table 5 that the employees have been much satisfied through customer service provided by banks in respect of the variables, quick response (61.61) mean scores followed by building relationship (61.38), innovative service delivery (47.20), good rapport (41.74), and financial security (38.07) in the order of priority. Hence it can be concluded that as bank is quick in responding to all its queries the employees are much satisfied with the service offered by Banking Industry.

The distribution of employees on the basis of meeting attended by them is given in Table 6.

It is inferred from Table 6 that, out of the total employees, 83.64 per cent said that they had attended the meeting conducted by banks, while the remaining 16.36 per cent said that they had not attended the meeting.

*Ho: there is no significant difference among the age of the employees with regard to the opinion of CRM strategy*. To test the above hypothesis one-way analysis of the variance is used (see Table 7).

Table 7 depicts that there is a significant difference among age with regard to the opinion of CRM strategy and hence null hypothesis (Ho) is rejected. Hence it is concluded that CRM strategy in Banking Industry has significant difference among age.

*Ho: there is no significant difference among the occupational status of the employees and the opinion of CRM strategy in Banking Industry*. To test the above hypothesis one-way analysis of the variance is used (see Table 8).

Table 8 shows that there is a significant difference among the occupational status of the employees with regard to the opinion of Banking Industry strategy and hence null hypothesis (Ho) is rejected. Hence it is concluded that CRM strategy in banks has significant difference among occupational status.

*Ho: there is no significant difference among the category of customers of the employees about the opinion of CRM strategy in Banking Industry*. To test the above hypothesis one-way analysis of the variance is used (see Table 9).

It is inferred from Table 9 that there is no significant difference among the category of customers approaching the employees with regard to the opinion of CRM strategy and hence null hypothesis (Ho) is accepted. Hence it is concluded that CRM strategy in banks does not have significant difference among category of customers.

The distribution of employees with regard to solving the problem of the account holders is given in Table 10.

Table 10 clearly shows that out of the total employees all of them said that they would solve the problem of the account holders.

### 7.2. How They Solve the Problem of the Account Holders

The most important ways by which the problem of the account holders is solved by the employees are classified into clearing the doubts of the account details, giving advice at the time of facing the problems, verifying all loan documents, providing moral support at all levels, and settling the grievance as quickly as possible. The distribution of employees on the basis of how they solve the problems of the account holders using KS Test is listed in Tables 11, 12, and 13.

#### 7.2.1. KS Test

For the purpose of analysing whether there is any difference in the importance of ratings given by the employees on various statements, the hypotheses have been formulated. The hypothesis has been tested by the researcher with the help of Kolmogorov-Smirnov test (here known as KS Test).

 Formula *D* = *O* − *E*
 
*D* refers to calculated value;
 
*O* refers to cumulative observed proportion;
 
*E* refers to cumulative expected proportion.

#### 7.2.2. Testing of Hypotheses

To assess the employees opinion regarding solving the problem of account holder in banks five statements, namely, clearing the doubts of the loan details, giving advice at the time of facing the problems, verifying all loan documents, providing moral support at all levels to them, and settling the grievances of the account holders as quickly as possible have been used and hypotheses are framed and tested by applying KS Test.

Cumulative observed proportion is calculated on the basis of observed frequency, that is, observed number. The total number of employees is 110. About 110 employees have given their opinion for gradation “Strongly Agree.” In the case of first statement the observed properties are calculated by dividing 60 of total employees. The resultant value (0.60) helps us to grade the observed properties. For all gradations, the same method of calculation is followed. On the basis of observed proportion, cumulative observed proportion is calculated.

Cumulative expected proportion is calculated on the basis of expected proportion. Since there are five gradations, each gradation (i.e., 0.20) is assigned as expected proportion. On the basis of expected proportion, the cumulative expected proportion is calculated.

For each gradation, the difference between cumulative observed proportion and cumulative expected proportion is calculated. The largest difference will be taken as calculated value. The calculated value is compared with the table value (Tables 11, 12, and 13).

If the calculated value is greater than the table value, the null hypothesis is rejected. On the other hand if the calculated value is less than the table value, the null hypothesis is accepted. 

*Result*

 Calculated value: (largest difference) = 0.60.
 The table value at 95 per cent confidence level = $1.36/\sqrt{110}$ = 0.13.

As the calculated value (0.60) is greater than the table value (0.13), the null hypothesis is rejected. Hence, there is difference in the importance of ratings given by the employees in clearing the doubts of the account and loan details in banks in Madurai district. 

*Result*

 Calculated value: (largest difference) = 0.40.
 The table value at 95 per cent confidence level = $1.36/\sqrt{110}$ = 0.13.

As the calculated value (0.40) is greater than the table value (0.13), the null hypothesis is rejected. Hence, there is difference in the importance of ratings given by the employees in giving advice at the time of facing the problems in banks at Madurai district.

The employees are asked to give their opinion at five-point scale ranging from “Strongly Agree” to “Strongly Disagree” for the following statements and the mean, standard deviation and coefficient of variation is given in Table 13.

### 7.3. Opinion about CRM System Is an Integral Part of the Work in Banks

“It is fully accepted that the CRM system is an integral part of work in bank”. The researcher has identified nine statements to study the opinion about the CRM systems as an integral of part of work in bank.

#### 7.3.1. Coefficient of Variations

The opinion of the employees about the CRM system as an integral part of the work in banks has been analysed through mean value ($\overset{-}{X}$), standard deviation (*σ*), and coefficient of variation. Table 13 shows the details.

Table 13 explicates that “CRM helps to build customer loyalty” has got 4.97 as mean value, 0.16 as standard deviation, and 3.22 as coefficient of variation, “CRM enhances customer loyalty” has got 4.74 as mean value, 0.50 as standard deviation, and 10.55 as coefficient of variation, “CRM can attract new customers” has got 4.77 as mean value, 0.46 as standard deviation, and 9.64, “CRM Boost Customer's Confidence” and “CRM Benefits industry performance and productivity” have got 4.02 as mean values, 0.13 as standard deviation, and 3.23 as coefficient of variations followed by “CRM objective is to frame customer database,” “CRM is undertaken by employee to approach customers,” Existence of CRM practices throughout all the level, and so on. Hence it is concluded that the opinion of the employees regarding the CRM System is an integral part of their work in banks.

## 8. Summary of Findings and Recommendations

This study provides an in-depth appraisal of stress existing in the banks positioned in the Madurai district of Tamil Nadu, India. The results of a range of tests have proven that pressure is invariably in audience everywhere in the bank organization. Bank staff are subjected to mounting levels of stress. The work situation staggers under the influence of stress and thereby is principal to a fruitless environment. In addition, interpersonal work connection is also under fire as the test outcome shows a decline in assistant support in the banks. Irrespective of the type, that is, management owned public sector banks or private banks, employees working under both sectors face extensive stress. It has also been found that the work experience and marital status have a collision on stress in workplace. Entertainingly, though the marital status adds its own weight on strain, the size of family does not have any endproduct on stress. The ever-escalating menace of traffic congestion in today's roads contributes to the stress in the work environment. The tests have proved that the distance from home to headquarters played a momentous role in inducing stress. The coworker and controller support were poor in the bank under cram. The results revealed that the human resources face considerable heaviness because of the way out support from their colleagues.

The art of coping with the stress using various strategies is the need of the day. It should be unwritten that the failure to protect the human being assets will lead to a calculated decay of a working culture. Banks should habitually organize destressing encampment to help the employees relieve their stress. The human resources have to be educated about the stress and its pessimistic impact on the family and work atmosphere. Stress buster techniques have to be educated to the employees. The secretarial setup has to be effectively redesigned so as to remove more work with little endeavor. The role of every employee should be defined clearly, so that role conflicts are null and void. Every major bank with more than 50 human resources shall have a separate space allotted for meditation room and a minipower fitness center. The people shall be encouraged to formulate use of these services and the banks can also think about introducing incentives to those people who use them commonly. Frequent get-together should be planned to bridge the gap between the supervisors and employees. This creates stronger bonds among coworkers.

## 9. Conclusion

Stress has practically invaded our life in the contemporary 21st century. We can never envisage a superlative world that does not have the part of strain in its day to day life. The bank exchange is the very hard and boring process; when compared to other areas it gives more stress than any other present surroundings. The character of employee obligation forms the vertebral column of any commerce and is inescapably responsible for administration of unbeaten business. So, the issue of job nervous tension has to be addressed with due concern and consideration. It has to be determined to improve the efficiency at workplace. In the present study we have addressed only a few demographic variables with the mention of the stress and analyze the various impact of coworker support and superintendent support to the employees. More delving into this subject can be taken up in the future so as to improve the capacity of the psychotherapy.

## Figures and Tables

**Table 1:** Know about CRM strategy of banking sector.

Sl. number	Rank	1	2	3	4	5	Total	Total Score	Average	Rank
	Scale how do they know about CRM strategy	75	60	50	40	25	110			
1	Through training									
	*f*	32	44	13	9	12	110	6350	57.73	II
	*fx*	2400	2640	650	360	300				

2	Through journals and magazines									
	*f*	5	13	56	32	4	110	5335	48.5	III
	*fx*	375	780	2800	1280	100				

3	Orientation given by officials									
	*f*	53	15	4	30	8	110	6475	58.86	I
	*fx*	3975	900	200	1200	200				

4	Through colleagues									
	*f*	8	29	14	36	23	110	5055	45.95	IV
	*fx*	600	1740	700	1440	575				

5	Personal experience									
	*f*	12	9	23	3	63	110	4285	38.95	V
	*fx*	900	540	1150	120	1575				

	Total	110	110	110	110	110				

Source: computed.

**Table 2:** Category of customers approaching.

Sl. number	Category	Number of employees	Percentage to total
1	Farmers	19	17.27
2	Businessmen	35	31.82
3	Professionals	40	36.36
4	Govt. employees	1	0.91
5	Private employees	15	13.64

	Total	110	100.00

Source: primary data.

**Table 3:** At which stage the customers are approaching.

Sl. number	Particulars	Number of employees	Percentage to total
1	At the time of opening new account and long term deposit	37	33.64
2	At the time of new account and long term deposit	2	1.82
3	At the time of closing the deposit and settlement	7	6.36
4	At the time of closing the deposit in middle	1	0.91
5	At the time of getting loan	30	27.27
6	At the time of transfer of money in account	2	1.82
7	At the time of loan repayment	31	28.18

	Total	110	100.00

Source: primary data.

**Table 4:** Satisfaction with customer services provided by Banking Industry.

Sl. number	Particulars	Number of employees	Percentage to total
1	Yes	84	76.36
2	No	26	23.64

	Total	110	100.00

Source: primary data.

**Table 5:** How you are satisfied with the services provided by banks.

Sl. number	Rank	1	2	3	4	5	Total	Total Score	Average	Rank
	Scale satisfaction with customer services provided by LIC	75	60	50	40	25	110			
1	Quick response									
	*f*	41	45	5	16	2	109	6715	61.61	I
	*fx*	3075	2700	250	640	50				

2	Innovative service delivery									
	*f*	3	3	68	31	4	109	5145	47.20	III
	*fx*	225	180	3400	1240	100				

3	Building relationship									
	*f*	60	14	1	30	4	109	6690	61.38	II
	*fx*	4500	840	50	1200	100				

4	Good rapport									
	*f*	3	25	14	30	37	109	4550	41.74	IV
	*fx*	225	1500	700	1200	925				

5	Financial security									
	*f*	2	22	21	2	62	110	4150	38.07	V
	*fx*	150	1320	1050	80	1550				

	Total	110	110	110	110	110				

Source: computed.

*Note*. *x*: scale value; *f*: number of employees; *fx*: score.

**Table 6:** Attended the meeting of banks.

Sl. number	Particulars	Number of employees	Percentage to total
1	Yes	92	83.64
2	No	18	16.36

	Total	110	100.00

Source: primary data.

**Table 7:** ANOVA among age of the employees about the opinion of CRM strategy in Banking Industry in Madurai District.

Age group	Sum of squares	d.f.	Mean square	*F*	Statistical inference
Between group	1.325	4	0.331	3.432	0.011 *p* < 0.05 Significant
Within group	10.138	105	0.097		

Total	11.464	109			

Source: computed data.

**Table 8:** ANOVA among occupational status of employees about the opinion of CRM strategy in Banking Industry in Madurai District.

Age group	Sum of squares	d.f.	Mean square	*F*	Statistical inference
Between group	6.275	4	1.569	2.548	0.044 *p* < 0.05 Significant
Within group	64.643	105	0.616		

Total	70.918	109			

Source: computed data.

**Table 9:** ANOVA among the categories of customers approaching employees about the opinion of CRM strategy in Banking Industry in Madurai District.

Age group	Sum of squares	d.f.	Mean square	*F*	Statistical inference
Between group	4.462	4	1.116	1.763	0.142 *p* > 0.05 Not significant
Within group	66.456	105	0.633		

Total	70.918	109			

Source: computed data.

**Table 10:** Solve the problem of the account holders.

Sl. number	Particulars	Number of employees	Percentage to total
1	Yes	110	100.00
2	No	—	—

	Total	110	100.00

Source: primary data.

**Table 11:** Clearing the doubts of the account and loan details, KS Test.

Sl. number	Opinion	Observed number	Observed proportion	Cumulative observed proportion	Expected proportion	Cumulative expected proportion	*D* = *O* − *E*
1	Strongly agree	45	0.41	0.41	0.20	0.20	0.21
2	Agree	65	0.59	1.00	0.20	0.40	0.60
3	No opinion	0	0.00	1.00	0.20	0.60	0.40
4	Disagree	0	0.00	1.00	0.20	0.80	0.20
5	Strongly disagree	0	0.00	1.00	0.20	1.00	0.00

**Table 12:** Giving advice at the time of facing the problems, KS Test.

Sl. number	Opinion	Observed number	Observed proportion	Cumulative observed proportion	Expected proportion	Cumulative expected proportion	*D* = *O* − *E*
1	Strongly agree	1	0.01	0.01	0.20	0.20	−0.19
2	Agree	66	0.60	0.61	0.20	0.40	0.21
3	No opinion	43	0.39	1.00	0.20	0.60	0.40
4	Disagree	0	0.00	1.00	0.20	0.80	0.20
5	Strongly disagree	0	0.00	1.00	0.20	1.00	0.00

Source: computed data.

**Table 13:** Opinion about the CRM system in an integral part of the work in banks.

Sl. number	Opinion of employees	$\overset{-}{X}$	*σ*	C.V
1	CRM enhances customers loyalty	4.74	0.50	10.55
2	Existence of CRM practices throughout all the levels	3.44	0.52	15.12
3	CRM is undertaken by employee to approach customers	3.80	0.40	10.53
4	CRM objective is to increase better relationship among the account holders	3.85	0.45	11.69
5	CRM objective is to frame customer database	2.87	0.53	18.47
6	CRM can attract new customers	4.77	0.46	9.64
7	CRM helps to build customer loyalty	4.97	0.16	3.22
8	CRM boosts customer's confidence	4.02	0.13	3.23
9	CRM benefits industry performance and productivity	4.02	0.13	3.23

Source: computed data.
